# Scarlet Fever Upsurge in England and Molecular-Genetic Analysis in North-West London, 2014

**DOI:** 10.3201/eid2206.151726

**Published:** 2016-06

**Authors:** Claire E. Turner, Marta Pyzio, Bonita Song, Theresa Lamagni, Margie Meltzer, J. Yimmy Chow, Androulla Efstratiou, Sally Curtis, Shiranee Sriskandan

**Affiliations:** Imperial College London, London, UK (C.E. Turner, M. Pyzio, S. Sriskandan);; Imperial College Healthcare NHS Trust, London (B. Song, S. Curtis);; Public Health England, London (T. Lamagni, M. Meltzer, J.Y. Chow, A. Efstratiou)

**Keywords:** scarlet fever, Streptococcus pyogenes, bacteria, streptococci, antimicrobial resistance, superantigens, genotype, respiratory infections, notifications, London, England

## Abstract

Scarlet fever notifications surged across the United Kingdom in spring 2014. Molecular epidemiologic investigation of *Streptococcus pyogenes* infections in North-West London highlighted increased *emm*4 and *emm*3 infections coincident with the upsurge. Unlike outbreaks in other countries, antimicrobial resistance was uncommon, highlighting an urgent need to better understand the drivers of scarlet fever activity.

An unprecedented rise in scarlet fever occurred in England in spring 2014, with >13,000 notifications, for an overall population rate of 24.5/100,000 persons (*1*,*2*). We analyzed clinical notification data for North-West London (population ≈1,900,400) during 2009–2014 and determined *emm* genotypes of *Streptococcus pyogenes* causing upper respiratory tract (URT) infections during 2009–2014. We focused on peak periods of scarlet fever notification.

## The Study

During weeks 10–20 (March–May) 2014, scarlet fever notifications in North-West London increased 3–8-fold compared with the same period in previous years (Figure, panel A). Although Health Protection regulations in England require clinicians to report suspected cases of scarlet fever, molecular surveillance of noninvasive *S. pyogenes* is not feasible because testing for *S. pyogenes* is not routinely advised for patients with a sore throat in the United Kingdom (*3*). Nonetheless, a limited number of URT swab specimens are submitted by clinicians for culture. Since 2009, we have stored all *S. pyogenes* URT isolates identified in our West London diagnostic laboratory, which serves a population of ≈2 million, overlapping with the North-West London region.

**Figure:**
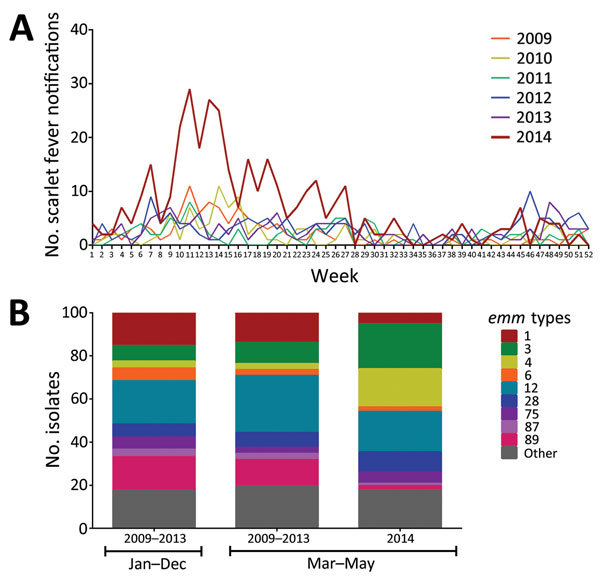
Increase in North-West London scarlet fever notifications and association with *emm*4 and *emm*3, 2014. A) Weekly scarlet fever notifications in North-West London during 2009–2014. During weeks 10–20 (March–May) 2014, the number of notifications substantially increased. B) *emm* genotyping of 404 upper respiratory tract *Streptococcus pyogenes* isolates. Isolates were available from March 2009 through May 2014, inclusive. A total of 308 isolates were from 2009–2013; however, of these, 134 were from 2009, and 174 were from 2010–2013, reflecting a fall in submission rates and affecting the availability of strains for study. Thus, isolates from 2009–2013 were considered a single group (January–December) and the following *emm-*types were identified: *emm*12 20% (62/308), *emm*89 16% (48/308), *emm*1 15% (47/308), *emm*3 7% (22/308), *emm*28 6% (19/308), *emm*4 3% (10/308). A similar pattern was observed during March–May in 2009–2013 (n = 72). A total 96 isolates were available from 2014, all of which were from March–May, submitted following alerts to clinicians regarding scarlet fever activity. During March–May 2014, isolates typed as *emm*4 increased significantly, from 3% (2/72) to 17% (17/96), (χ^2^_(1df)_ = 9.478. p = 0.0021). A borderline significant increase occurred in *emm*3, from 10% (7/72) in 2009–2013 to 20% (20/96) in 2014 (χ^2^_(1df)_ = 3.766, p = 0.0523). This constituted a 3-fold increase in *emm*3 and *emm*4 combined, from 13% in 2009–2013 to 37% in 2014 (March–May).

Molecular testing, with standard DNA extraction, *emm* typing, and superantigen typing methods (*4*), was performed on all 404 viable *S. pyogenes* URT isolates identified. Of all isolates obtained from March through May 2009–2013 (n = 72), predominant *emm* genotypes were *emm*12 (26%), *emm*89 (13%), and *emm*1 (14%) (Figure, panel B). These proportions were almost identical to proportions for all 308 isolates obtained throughout 2009–2013 (Figure, panel B). In contrast, during March–May 2014 (n = 96), dominating *emm* types changed, with a borderline significant increase in *emm*3 (from 10% in March–May 2009–2013 to 20% in 2014; χ^2^_(1df)_ = 3.766, p = 0.0523), and a significant >5-fold increase in *emm*4 (from 3% to 17%; χ^2^_(1df)_ = 9.478, p = 0.0021). Among 96 URT samples submitted in March–May 2014, a total of 42 were from children ages ≤5 years. *Emm*4 was significantly associated with age ≤5 years (χ^2^_(1df)_ = 6.046, p = 0.0139), and the rise was largely attributable to disease in this age group (12/17 *emm*4 isolates).

Isolates from March–May 2014 were categorized by at least one of the following clinical features (provided by submitting physician): 1) tonsillitis, pharyngitis, or sore throat and no mention of scarlet fever (n = 44); 2) any mention of scarlet fever, regardless of other information (n = 16); 3) any other illness (n = 6); and 4) no details provided (n = 30). The 16 scarlet fever–associated isolates were limited to patients ages 1.25–11 years, a significant proportion of whom were ≤5 years (12/16; χ^2^_(1df)_ = 7.619, p = 0.0058); 7/16 were *emm*3 and 3/16 were *emm*4. The remainder were *emm*12 (3), *emm*28 (2), and *emm*87 (1). On the basis of these limited data, *emm*3 was significantly associated with scarlet fever in 2014 (χ^2^_(1df)_ = 5.964, p = 0.0146).

Clinical data were not collected in earlier years routinely, although in 2009 a total of 3/3 isolates from scarlet fever case-patients were *emm*3. Scarlet fever–associated *emm*4 strains from 2014 (n = 3) carried superantigens *speC*, *ssa,* and *smeZ*; the same superantigen profile was found in *emm*4 strains from patients for whom scarlet fever was not mentioned (n = 14). All 7 scarlet fever–associated *emm*3 strains carried *speA*, *ssa*, *speG,* and a known mutation in *smeZ*.

Antimicrobial drug resistance was identified in 10/96 URT isolates from 2014; however, none of these isolates were associated with scarlet fever and none were *emm*4. Erythromycin resistance was found in 2/20 non–scarlet fever *emm*3 isolates, in combination with clindamycin resistance in 1 isolate.

## Conclusions

An increase in *emm*3 and *emm*4 *S. pyogenes* URT isolates was detected in North-West London, during the period in 2014 when scarlet fever notifications peaked. The increase in *emm*4 infections was also found predominantly in 4- to 5-year-old children, the group we and others found to be most at risk for scarlet fever (*1*). The percentage of children 4 years old in North-West London (an urban population) is similar to the national average of 1.3%; therefore, our findings are probably relevant to the rest of the United Kingdom.

*Emm*4 isolates accounted for only 3/16 cases in which scarlet fever was mentioned, although, because of the study’s retrospective nature and paucity of clinical data supplied, we cannot dismiss the possibility that other *emm*4 isolates were also associated with scarlet fever. On the basis of the limited analysis of isolates from infections in which scarlet fever was mentioned, we found an association between scarlet fever and *S. pyogenes* *emm*3 strains.

The results of our historical comparison must be interpreted with caution; obtaining swab samples from patients with URT infections in England is not routine. Thus, the 2009–2013 samples may reflect persistent infections, in contrast to 2014 samples, when clinicians were encouraged to submit swab specimens for scarlet fever case-patients. Furthermore, the number of strains available for *emm* typing was limited. Nonetheless, this was the only collection of strains available to us that permitted historical comparison.

Both *emm*3 and *emm*4 *S. pyogenes* strains have been associated with scarlet fever (*5*). In the Far East, *emm*1 and *emm*4 isolates were the leading causes of scarlet fever in the late 1990s (*6*), although more recently, antimicrobial drug–resistant *emm*12 *S. pyogenes* has dominated in this region (*7*–*9*). We found that the proportion of *emm*12 isolates fell during the scarlet fever surge and found no antimicrobial drug resistance among *emm*3 or *emm*4 isolates associated with scarlet fever.

*Emm*4 isolates are associated with pharyngitis in children (*10*,*11*); these isolates are entirely acapsular, a phenotype linked to enhanced adhesion to surfaces (*12*). Whether this characteristic can increase persistence and transmission is unknown. Surges in scarlet fever are believed to require a population susceptible to pharyngeal infection with specific strain types and specific superantigens. Both *emm*3 and *emm*4 strains in our study possessed 2 prophage-associated superantigens, either SPEA and SSA, or SPEC and SSA. Although these toxin genes were found in *emm*3 and *emm*4 strains not associated with scarlet fever, the probability of triggering scarlet fever may be enhanced through production of 2 such superantigens. An association between these superantigens and scarlet fever has been reported (*13*).

Periodic increases in scarlet fever are well recognized, although the magnitude of the upsurge in the United Kingdom was unexpected. Consultation rates for sore throat diminished in the 1990s (*14*), and the 2008 UK national guidelines advise against diagnostic testing and recommend a policy of nonprescribing or delayed prescribing for sore throat when the Centor score is <3 (*3*). These recommendations contrast with those of North America and of some European countries (*15*). Whether exceeding a threshold level of community *S. pyogenes* transmission is required for such a marked upsurge is unclear; increased scarlet fever activity was not reported elsewhere in Europe, to our knowledge. Apart from natural fluctuations in population immunity, emergence of hypertransmissible lineages, acquisition of novel phage-encoded toxins, or antimicrobial drug resistance may contribute to scarlet fever surges (*6*,*7*). Notably, isolates we found associated with scarlet fever were not resistant to common antimicrobial agents.

As part of the national response, clinicians were advised to treat scarlet fever to minimize complications and reduce transmission. Whether use of more refined molecular diagnostics could assist future community prevention and management of *S. pyogenes* infection will require careful evaluation. Increased scarlet fever activity has continued in England in 2015 and 2016, underscoring the need for ongoing surveillance and further investigation. 

## References

[1] Guy R, Williams C, Irvine N, Reynolds A, Coelho J, Saliba V, Increase in scarlet fever notifications in the United Kingdom, 2013/2014. Euro Surveill. 2014;19:20749. 10.2807/1560-7917.ES2014.19.12.20749 24698137

[2] Public Health England. Group A streptococcal infections: seasonal activity, 2014/15. Health Protection Report. 2014;8 [cited 2015 Sep 28]. https://www.gov.uk/government/uploads/system/uploads/attachment_data/file/377520/hpr4414_SF.pdf

[3] National Institute for Health and Care Excellence. Respiratory tract infections: antibiotic prescribing. Prescribing antibiotics for self-limiting respiratory tract infections in adults and children in primary care (NICE guideline), July 2008 [cited 2015 Sep 28]. https://www.nice.org.uk/guidance/CG69/chapter/1-Guidance 21698847

[4] Turner CE, Dryden M, Holden MT, Davies FJ, Lawrenson RA, Farzaneh L, Molecular analysis of an outbreak of lethal postpartum sepsis caused by *Streptococcus pyogenes.* J Clin Microbiol. 2013;51:2089–95. 10.1128/JCM.00679-13 23616448 PMC3697669

[5] Perks EM, Mayon-White RT. The incidence of scarlet fever. J Hyg Camb. 1983;91:203–9. 10.1017/S0022172400060204 6358344 PMC2129386

[6] Yan JJ, Liu CC, Ko WC, Hsu SY, Wu HM, Lin YS, Molecular analysis of group A streptococcal isolates associated with scarlet fever in southern Taiwan between 1993 and 2002. J Clin Microbiol. 2003;41:4858–61. 10.1128/JCM.41.10.4858-4861.2003 14532243 PMC254326

[7] Tse H, Bao JY, Davies MR, Maamary P, Tsoi HW, Tong AH, Molecular characterization of the 2011 Hong Kong scarlet fever outbreak. J Infect Dis. 2012;206:341–51. 10.1093/infdis/jis362 22615319 PMC4125623

[8] Chiou CS, Wang YW, Chen PL, Wang WL, Wu PF, Wei HL. Association of the shuffling of *Streptococcus pyogenes* clones and the fluctuation of scarlet fever cases between 2000 and 2006 in central Taiwan. BMC Microbiol. 2009;9:115. 10.1186/1471-2180-9-115 19486515 PMC2697166

[9] Luk EYY, Lo JYC, Li AZL, Lau MCK, Cheung TKM, Wong AYM, Scarlet fever epidemic, Hong Kong, 2011. Emerg Infect Dis. 2012;18:1658–61. 10.3201/eid1810.111900 23018120 PMC3471614

[10] Jaggi P, Tanz RR, Beall B, Shulman ST. Age influences the *emm* type distribution of pediatric group A streptococcal pharyngeal isolates. Pediatr Infect Dis J. 2005;24:1089–92. 10.1097/01.inf.0000190023.89759.96 16371871

[11] Shulman ST, Tanz RR, Dale JB, Beall B, Kabat W, Kabat K, Seven-year surveillance of North American pediatric group A streptococcal pharyngitis isolates. Clin Infect Dis. 2009;49:78–84. 10.1086/599344 19480575

[12] Turner CE, Abbott J, Lamagni T, Holden MT, David S, Jones MD, Emergence of a new highly successful acapsular group A *Streptococcus* clade of genotype *emm*89 in the United Kingdom. MBio. 2015;6:e00622. 10.1128/mBio.00622-15 26173696 PMC4502227

[13] Silva-Costa C, Carrico JA, Ramirez M, Melo-Cristino J. Scarlet fever is caused by a limited number of *Streptococcus pyogenes* lineages and is associated with the exotoxin genes *ssa, speA* and *speC.* Pediatr Infect Dis J. 2014;33:306–1. 10.1097/INF.0000000000000088 24168973

[14] Ashworth M, Latinovic R, Charlton J, Cox K, Rowlands G, Gulliford M. Why has antibiotic prescribing for respiratory illness declined in primary care? A longitudinal study using the General Practice Research Database. J Public Health (Oxf). 2004;26:268–74. 10.1093/pubmed/fdh160 15454595

[15] Chiappini E, Regoli M, Bonsignori F, Sollai S, Parretti A, Galli L, Analysis of different recommendations from international guidelines for the management of acute pharyngitis in adults and children. Clin Ther. 2011;33:48–58. 10.1016/j.clinthera.2011.02.001 21397773

